# Genotypic Variation and Mixtures of Lyme *Borrelia* in *Ixodes* Ticks from North America and Europe

**DOI:** 10.1371/journal.pone.0010650

**Published:** 2010-05-14

**Authors:** Chris D. Crowder, Heather E. Matthews, Steven Schutzer, Megan A. Rounds, Benjamin J. Luft, Oliver Nolte, Scott R. Campbell, Curtis A. Phillipson, Feng Li, Ranga Sampath, David J. Ecker, Mark W. Eshoo

**Affiliations:** 1 Ibis Biosciences, Carlsbad, California, United States of America; 2 Suffolk County Department of Health Services, Yaphank, New York, United States of America; 3 Department of Medicine, State University of New York at Stony Brook, Stony Brook, New York, United States of America; 4 Laboratory of Dr. Brunner, Constance, Germany; 5 Department of Medicine, University of Medicine and Dentistry of New Jersey, Newark, New Jersey, United States of America; BMSI-A*STAR, Singapore

## Abstract

**Background:**

Lyme disease, caused by various species of *Borrelia*, is transmitted by *Ixodes* ticks in North America and Europe. Studies have shown the genotype of *Borrelia burgdorferi* sensu stricto (s.s.) or the species of *B. burgdorferi* sensu lato (s.l.) affects the ability of the bacteria to cause local or disseminated infection in humans.

**Methodology/Principal Findings:**

We used a multilocus PCR electrospray mass spectrometry assay to determine the species and genotype *Borrelia* from ticks collected in New York, Connecticut, Indiana, Southern Germany, and California and characterized isolates from parts of the United States and Europe. These analyses identified 53 distinct genotypes of *B. burgdorferi* sensu stricto with higher resolution than ospC typing. Genotypes of other members of the *B. burgdorferi* sensu lato complex were also identified and genotyped including *B. afzelii, B. garinii, B. lusitaniae, B. spielmanii, and B. valaisiana*. While each site in North America had genotypes unique to that location, we found genotypes shared between individual regions and two genotypes found across the United States. Significant *B. burgdorferi* s.s. genotypic diversity was observed between North America and Europe: only 6.6% of US genotypes (3 of 45) were found in Europe and 27% of the European genotypes (3 of 11) were observed in the US. Interestingly, 39% of adult *Ixodes scapularis* ticks from North America were infected with more than one genotype of *B. burgdorferi* s.s. and 22.2% of *Ixodes ricinus* ticks from Germany were infected with more than one genotype of *B. burgdorferi* s.l.

**Conclusions/Significance:**

The presence of multiple *Borrelia* genotypes in ticks increases the probability that a person will be infected with more than one genotype of *B. burgdorferi*, potentially increasing the risks of disseminated Lyme disease. Our study indicates that the genotypic diversity of *Borrelia* in ticks in both North America and Europe is higher then previously reported and can have potential clinical consequences.

## Introduction

*Borrelia burgdorferi* sensu stricto is a bacterial spirochete that is one of the causative agents of Lyme disease[1], [2], [3]. Human risk for the disease may be dependent on a complex interaction between environment and vector [1] with transmission of the resultant bacterial genotypes an essential determinant of disease.

*B. burgdorferi* is transmitted by *Ixodes* ticks: *I. scapularis and I. pacificus* in North America and *I. ricinus* in Europe [2], [3], [4]. The tick often acquires the spirochetes during its larval stage by feeding on an infected host, usually small rodents [5]. After molting, the nymphs feed on small mammals or birds; adults primarily on deer. It is during these feedings as a nymph or adult that the spirochetes from an infected tick can be transmitted to a new host. Humans, although not part of the natural enzootic cycle, can become infected with *B. burgdorferi* after being bitten by an infected tick, primarily by nymphs due to their small size. Lyme disease in humans may be localized to the site of the tick bite and is often characterized by the almost pathognomonic bull's-eye-like lesion called erythema migrans (EM). In other cases, the infection may disseminate and produce systemic pathology affecting other skin sites, the nervous system, the joints, or the heart [6], [7], [8], [9].

In the past, researchers often classified *B. burgdorferi* s.s. by the type of outer surface protein C (ospC) expressed [10], [11]. There is a demonstrated correlation between ospC type and the rodent host [12], suggesting that some host species only carry *B. burgdorferi* s.s. expressing a certain ospC type. Studies have shown that patients who present with localized disease (i.e., a single bull's-eye lesion) have a small subset of ospC types relative to the ospC types found in ticks from the entire region. A smaller subset of ospC types has been found in patients with systemic disease, suggesting that the ospC type may be one marker for disease dissemination in humans [10].

Multilocus sequence typing (MLST) and multilocus variable-number tandem repeat (VNTR) analysis has been used to distinguish *B. burgdorferi* sensu stricto and sensu lato isolates and examine their evolutionary lineage [13], [14], [15], [16]. MLST has been shown to have a higher resolving power than other methods [15]. However, traditional MLST requires sequencing of each locus to define the genotype and cannot resolve mixtures of genotypes. Multilocus PCR followed by electrospray ionization mass spectrometry (PCR/ESI-MS) has been used to genotype a number of medically relevant organisms [17], [18], [19], [20], [21], [22] and to detect tick-borne pathogens in clinical specimens [23]. To efficiently identify the different species and genotypes of *B. burgdorferi* s.s. and s.l., we used multilocus PCR/ESI-MS genotyping assay to characterize *Borrelia* populations in individual ticks.

Using the PCR/ESI-MS genotyping assay on a diverse collection of culture isolates and tick extracts we identified 53 different genotypes of *B. burgdorferi* s.s.. Furthermore the assay was able to characterize members of the *B. burgdorferi* s.l. group to the species and genotype levels. Results of this analysis demonstrated significant *B. burgdorferi* s.s. genotypic diversity among different regions in the United States and even more diversity between North American and European strains. The PCR/ESI-MS genotyping assay further identified a high prevalence of ticks containing multiple *B. burgdorferi* genotypes in the US and Europe. Exposure to multiple *Borrelia* genotypes from a single tick bite may further increase the risk of disseminated Lyme disease.

## Materials and Methods

### Multilocus PCR/ESI-MS genotyping assay

The PCR/ESI-MS genotyping assay employs eight PCR primer pairs that target seven *Borrelia* genes (Table 1). The primers are designed to amplify and distinguish all known *Borrelia* species by targeting conserved regions of DNA that amplify variable regions. All primers used in this study had a thymine nucleotide at the 5′-end to minimize the addition of non-templated adenosines during amplification with *Taq* polymerase [24]. An internal positive control (calibrant) made from synthetic DNA (BlueHeron Biotechnology, Bothell, WA) was included in each PCR reaction at 50 copies per PCR reaction. The calibrant sequence contains a five-base pair (bp) deletion within the amplicon so that the calibrant amplicons can be resolved from the bacteria-derived amplicons; the calibrant serves as an internal positive PCR control and allows quantification of amplicons in the PCR reaction. Nucleic acid extract from individual ticks was added to each of eight wells of 96-well plates containing everything required for PCR except the template DNA. PCR was performed in a 40-µL reaction containing 1 µL nucleic acid, 1 unit of Immolase Taq polymerase (Bioline USA, Taunton, MA), 20 mM Tris (pH 8.3), 75 mM KCl, 1.5 mM MgCl_2_, 0.4 M betaine, 200 µM dATP, 200 µM dCTP, 200 µM dTTP (each dNTP from Bioline USA), and 200 µM ^13^C-enriched dGTP (Cambridge Isotope Laboratories, Andover, MA), 20 mM sorbitol (Sigma-Aldrich, St. Louis, MO), 2 µg/mL sonicated poly-A RNA (Sigma-Aldrich), 500 µg/mL of ultrapure BSA (Invitrogen, Carlsbad, CA), and 250 nM of each primer. The following PCR cycling conditions were used on an MJ Dyad 96-well thermocycler (Bio-Rad Inc., Hercules, CA): 95°C for 10 min, followed by 8 cycles of 95°C for 30 s, 48°C for 30 s, and 72°C for 30 s, with the 48°C annealing temperature increasing 0.9°C for each cycle. The PCR was then continued for 37 additional cycles of 95°C for 15 s, 56°C for 20 s, and 72°C for 20 s. The PCR cycle ended with a final extension of 2 min at 72°C followed by a 4°C hold. The cycled PCR plates were then processed on the Ibis Bioscience (Carlsbad, CA) T5000 ESI-MS system.

**Table 1:** Primers used in genotyping assay.

Primer Pair ID	Primer code	Gene target	Primer sequence (5′-3′)
BCT3511	BCT8229F	*gyrB*	TGCATTTGAAAGCTTGGCATTGCC
	BCT8230R		TCATTTTAGCACTTCCTCCAGCAGAATC
BCT3514	BCT8235F	*rpoC*	TTTGGTACCACAAAGGAATGGGA
	BCT8236R		TGCGAGCTCTATATGCCCCAT
BCT3515	BCT8237F	*rplB*	TCCACAAGGTGGTGGTGAAGG
	BCT8238R		TCGGCTGTCCCCAAGGAG
BCT3516	BCT8239F	*leuS*	TCATGTTGGTCATCCGGAAGG
	BCT8240R		TTGCATAACTTTCAGCAGGAAGTCC
BCT3517	BCT8241F	*flaB*	TGCTGAAGAGCTTGGAATGCA
	BCT8242R		TACAGCAATTGCTTCATCTTGATTTGC
BCT3518	BCT8243F	*ospC*	TGACGGTATTTTTATTTATATCTTGTAATAATTCAGG
	BCT8244R		TTTGCTTATTTCTGTAAGATTAGGCCCTTT
BCT3519	BCT8245F	*hbb*	TCGAATAATGTTATTGAGTTTAGATCTTTTGGTAC
	BCT8246R		TGGACGAAAATACGCAACATGATGATC
BCT3520	BCT8247F	*hbb*	TGTCTTTTCCAAGAAGACCAAAGGTTACTAA
	BCT8248R		TACCCTTAAGCTCTTCAAAAAAAGCATC

### ESI-MS and base composition analysis

The base composition of the PCR amplicons was determined by ESI-MS using the Ibis Biosciences T5000 system as previously described [21], [25], [26].

### *Borrelia* isolates and nucleic acid extraction

*Borrelia* isolates were characterized from nucleic acid extracted from cultured isolates, field-collected ticks, and a clinical specimen. Adult and nymphal *Ixodes* ticks were collected in New York, Connecticut, California, Indiana, and southern Germany (Table 2). The species of the tick was determined by a trained entomologist. Ticks from the United States were collected through use of a drag cloth. Total nucleic acids were extracted from ticks from North American and from skin biopsy as previous described [27]. Briefly, the specimens were homogenized by bead-beating with zirconia/yttria beads (Glen Mills, Clifton, NJ) and the nucleic acids were extracted using a modified Qiagen MinElute Virus spin kit protocol. Nucleic acids were eluted in AVE buffer (Qiagen) and stored at −80°C.

**Table 2:** Sample origin locations and prevalence of *Borrelia* infected ticks.

State	County	Collection Site	Sample type	Samples (n)	*Borrelia* infected (i)	*B. burgdorferi* s.s. Genotypes found
California	Marin	China Camp SP, Olompali SP	Adult	141	2	2
	Napa	Bothe SP, Skyline Regional Park	Adult	186	2	1
	Sonoma	Glen Ellen	Nymphal	44	3	3, 4
	Glenn	Elk Creek	Adult	69	0	
	Santa Cruz	Nisene Marks SP, Felton	Adult	64	0	
	Placer	Colfax, Foresthill, Lake Arthur, Drivers Flat	Adult	250	6	32, 34
	Lake	Clear Lake SP	Adult	128	2	
	Humbolt	Arcata	Adult	14	0	
	San Joaquin	Lodi	Adult	10	0	
	Mendocino	Russian Gulch SP	Adult	5	0	
	Del Norte	Patrick's Creek Six Rivers National Forest	Adult	15	0	
	Alameda	Oakland	Adult	22	0	
	Total		Ticks	948	15	
Connecticut	Fairfield	Bridgeport	Adult	99	67	1, 6, 7, 8, 10, 11, 12, 13, 22, 23, 33, 34, 36, 37, 38, 39, 40, 41, 42
Indiana	Pulaski	Tippecanoe River State Park	Adult	81	19	2, 10, 11, 22, 25, 26, 32, 34, 39
New York	Dutchess		Nymphal	74	11	31, 32
	Suffolk	Shelter Island, Connetquot, Mastic	Adult	179	109	1, 6, 7, 8, 10, 11, 12, 13, 15, 22, 23, 33, 34, 35, 40, 41, 44, 45, 46, 47, 48
			Nymphal	24	11	1, 11, 22, 23, 33, 41, 43, 53
	Westchester	Franklin D. Roosevelt State Park - Ben Luft	Adult	44	24	1, 6, 7, 10, 11, 15, 23, 25, 30, 32, 34, 44, 52
	Total			321	155	
Germany		Konstanz	unknown	178	34	9
NJ			skin biopsy	1	1	33
CDC			culture isolate	9	N/A	N/A
Stony Brook			culture isolate	79	N/A	N/A

Cultured isolates were provided by Barbara J. Johnson at the CDC in Ft. Collins, CO. DNA was extracted from these isolates by diluting the sample in water and heating to 95°C for 10 min. DNA from isolates previously characterized by their ospC type was provided by the laboratory of Benjamin Luft at Stony Brook University, NY [28].

*Ixodes ricinus* ticks were collected either in the area of Constance by flagging (Lake Constance, Southern Germany) or obtained from patients living in the southwestern parts of Germany. All ticks were visually identified as *I. ricinus* by an entomologist. DNA from large *Ixodes ricinus* ticks was obtained by slicing the ticks open using a sterile single-use scalpel blade and transferring the material to a 1.5 mL reaction tube, containing 200 µL QIAGEN's ATL-buffer (QIAamp DNA Mini Kit; QIAGEN, Hilden, Germany). Nymphs and larvae were transferred into 1.5 mL reaction tubes, containing 200 µL ATL-buffer and squeezed with a single use, sterile PP pistil (Carl Roth, Karlsruhe, Germany). The crude lysates were digested for a minimum of 3 h (nymphs and larvae) and a maximum overnight (adults) after addition of 20 µL Proteinase K. After brief centrifugation, supernatants were transferred to sterile 2.0 mL screw capped tubes (Sarstedt, Nümbrecht, Germany). DNA was extracted in a QIAcube instrument, applying the protocol “QIAamp DNA Mini–Blood or body fluid–Manual Lysis”, available from the QIAGEN homepage. The elution volume was set to 200 µL.

### Data analysis and interpretation

The basecount signature was used to determine the genotype of a *Borrelia*-containing specimen. If two isolates differed by a single base count they were considered to be unique genotypes. Primer pairs BCT3514 and BCT3515 were principally used to determine the species of *Borrelia* and the remaining primer pairs were used to genotype the organisms. When more than one allele was detected for any of the gene targets, the sample was considered to contain a mixture of *Borrelia burgdorferi* genotypes. If a mixture differed at more than one of the eight loci, we required a 1.5-fold difference in the amplitudes to assign the alleles to the major and minor contributors. When the mixtures were present at equal levels the genotypes could not be resolved.

## Results

### Multilocus PCR/ESI-MS genotyping of *Borrelia*

The multilocus PCR/ESI-MS genotyping assay was evaluated by testing 351 *Borrelia burgdorferi* s.l. positive specimens: 61 cultured isolates from North America and Europe, 256 *B. burgdorferi s.s.* infected field-collected ticks from North America and 34 ticks infected with *Borrelia* from Europe (Table 2). These analyses resulted in the identification of 53 distinct *B. burgdorferi* s.s. genotypes as shown in Table 3. Primer pairs BCT3514 and BCT3515 produced a consistent signature for *B. burgdorferi* s.s., but distinguish other members of the *B. burgdorferi* s.l. complex: *B. afzelii, B. garinii*, *B. valaisiana, B. andersonii, B. bissettii, and B. lusitaniae* (Table S1). The allelic diversity of the loci ranged from a high of 21 alleles for primer pair BCT3518 (targeting the *OspC* gene) to a low of four alleles for primer pair BCT3514, which was used to identify the *Borrelia* species. The multilocus PCR/ESI-MS genotyping assay identified nine genotypes of *B. afzelii* and four genotypes of *B. garinii*. All eight primer pairs detected a unique base count signature for *B. valaisiana, B. andersonii, B. bissettii, and B. lusitaniae*, but because there was only one isolate of each we could not demonstrate the assay's ability to genotype these species (Table 4). One sample of *B. spielmanii* was also detected on six of the eight primer pairs. Additionally, the assay was able to genotype *B. burgdorferi* directly from a skin biopsy from a patient presenting with erythema migrans. Results of this analysis showed the patient was infected with our genotype 33 (specimen NJ1, Table 3).

**Table 3:** *Borrelia burgdorferi* isolates and genotype.

Specimen ID^1^	Genotype	Specimen ID^1^	Genotype
109A, 160B, CA3, CT13, CT20, CT32, CT38, CT39*, CT45, NY5, NY13*, NY33, NY74, NY76, NY83, NY87, CT23*, CT6	**1**	MI418	**27**
CA1, CA2, IN9*	**2**	168a	**28**
CA92-0953, CA6	**3**	*Bol26*	**29**
CA92-1096, CA4, CA5, CA11	**4**	NY85*	**30**
NC92-0972	**5**	NY4, NY51	**31**
B31, 297, 136b, 132a, 163b, 132b, *IP2, PKa2, IP3, L65, H3, IP1, HB1, Lenz, HII, Ho, OEA11, 132A, Z11*, CT28*, CT42*, CT48, NY24, NY27, NY40, NY55, NY75*	**6**	CA8, IN6, IN12*, NY84, NY85*, IN9*	**32**
CT8, CT19, CT31, CT34, CT46, NY23*, NY48, NY73*	**7**	CT42*, NY2, NY58, NJ1	**33**
NC-2, JD1, CT11, NY20, NY22, NY25, NY45, NY46, NY53, NY59*, NY41*	**8**	CA7, IN10, CT6, CT24, NY10*, NY15*, NY32, NY57, NY71*, NY78	**34**
si-4, *217-5, Bol6, Z6, GER_204*	**9**	CA9, NY69	**35**
WI-MCI, IN5, CT41*, CT49*, NY50, NY70, NY79	**10**	CT30, NY1	**36**
IN2, IN8, CT4, CT10, CT12, CT16*, CT17*, CT21, CT23*, CT25, CT26, CT27, CT33, CT35, CT36*, CT37, CT40, NY8, NY16, NY18, NY29, NY34, NY36*, NY37, NY39, NY42*, NY44, NY47, NY52, NY64, NY66, NY72, CT39*, NY59*, CT16*, CT36*, NY36*, NY52, NY71*	**11**	CT18	**37**
94a, CT1, CT5, CT9, CT47, NY12, NY17, NY26, NY30, NY35, NY41*, NY65, NY67, NY68	**12**	CT29	**38**
CT14, CT44, NY54	**13**	CT41*, IN12*, IN1, IN11	**39**
*Bol12, Bol24, Lx36, ZS7, DK7, Y32, Y2, Y3, HY19, Y20, Y9*	**14**	NY13*, CT28*, CT17*	**40**
N40, *JS91, KL55, SD91, NP14*, 88a, *KI93*, GI71, NY28*, NY38, NY49*, NY56, NY60, NY82	**15**	CT2*, NY7*, NY15*	**41**
*Bol29, Bol30, MT30, KD13, SF3, KI29, BL31679, VR26*	**16**	CT50	**42**
*Z9*	**17**	NY14	**43**
*Bol27, Bol15, FR-93/1, Bol25, Bol2*	**18**	NY75*, NY10*	**44**
*VS219*	**19**	NY11*	**45**
*Y1, Y10, Ri9, Y14*	**20**	NY11*	**46**
*Ri5*	**21**	NY19	**47**
86b, 97b, IN3, CT2*, CT15*, NY7*, NY61*, NY63	**22**	NY28*	**48**
80a, 72a, CT3*, CT7, CT22, CT43, NY3, NY6, NY9, NY21, NY43, NY62, NY77, NY80	**23**	NY31	**49**
MI407	**24**	NY23*	**50**
156a, IN7, NY81*, NY86	**25**	NY42*	**51**
MI415, IN4*	**26**	NY73*	**52**
		CA10	**53**

**^1^Italicized samples are European in origin; unitalicized samples are United States in origin.**

*Denotes a mixture of more then one genotype.

**Table 4:** Other *Borrelia species* genotypes.

Species	Specimen ID^1^	Genotype
***Borrelia afzelii***	*ACA1 (UK-814), GER_169*	**1**
	*pKo, GER_106, GER_139* *	**2**
	*GER_033, GER_038*	**3**
	*GER_005* *	**4**
	*GER_005* *, *GER_139* *, *GER_176, GER_178* *, *GER_200*	**5**
	*GER_119, GER_178* *	**6**
	*GER_022, GER_116*	**7**
	*GER_125*	**8**
	*GER_004*	**9**
***Borrelia garinii***	*VSBM*	**1**
	*NT29 HS9*	**2**
	*GER_158*	**3**
	*GER_020*	**4**
***B. valaisiana***	*GER_143*	**1**
***B. andersonii***	*19857*	**1**
***B. bissettii***	*DN127*	**1**
***B. lusitaniae***	*PotiB1*	**1**
***B. spielmanii***	*GER_173*	**1**

Italicized samples are European in origin; non-italicized samples are United States in origin.

*Denotes a mixture of more then one genotype.

### Comparison of multilocus PCR/ESI-MS genotyping with *ospC* typing

A previously characterized collection of 52 isolates representing 18 ospC types were analyzed using the multilocus PCR/ESI-MS genotyping assay. For the 18 different ospC types examined, we observed 21 different multilocus PCR/ESI-MS genotypes. The results of this analysis are summarized in Table 5.

**Table 5:** Comparision of *ospC* typing vs. PCR/ESI-MS genotyping.

Strain	ospC Type	Genotype
B31, IP2, PKa2, IP3, Lenz, L65, HII, Ho, IP1, HB1, B31, 132a, 132b	A	6
MI415	B1	26
109a, 160b	B1	1
Bol12, Lx36, ZS7	B2	14
VS219	B2	19
JD1	C	8
SD91, NP14, N40, 88a	E	15
MI407	F	24
72a, 80a	G	23
MI411, 156a	H	25
86b, 97b	I	22
118a	J	28
297, OEA11, 136b, 163b	K	7
Y1, Y10	L	20
217-5, Bol6, Z6	L	9
MI418	N	27
Bol15, Bol25, Bol27, Fr-93/1	Q	18
Z9	S	17
94a	U	12
Bol29, Bol30	V	16
Ri5	W	21

### Characterization of *Borrelia burgdorferi* s.s. in *Ixodes* ticks

Nucleic acids were extracted from the ticks and screened for *B. burgdorferi* using primer pair BCT3515. A total of 501 *Ixodes scapularis* ticks (403 adults and 98 nymphs) were collected and analyzed from one site in Indiana (adults), five sites in New York (adults and nymphs), and one site in Connecticut (adults) during the 2008 and 2009 tick seasons. During the same years, 948 *Ixodes pacificus* ticks (904 adults and 44 nymphs) were collected and analyzed from 18 sites in California (Table 2). New York State had a *B. burgdorferi* infection rate of 59.6% in adult ticks and 45.8% in nymphs. It should be noted that the nymphal ticks obtained from Dutchess county NY were dead and dessicated at the time of testing, potentially affecting *B. burgdorferi* detections, and therefore were excluded from the infection rate determination. Connecticut had a higher infection rate with 67 of the 99 adult ticks positive for *B. burgdorferi* (67.7%). In Indiana 19 of 81 of adults ticks sampled (23.5%) were infected with *B. burgdorferi*. California had the lowest infection rate with 12 of 904 adult *I. pacificus* ticks and 3 of 44 nymphs (1.4% and 6.8% respectively) positive for *B. burgdorferi*, (Table 2).

**Figure 1:**
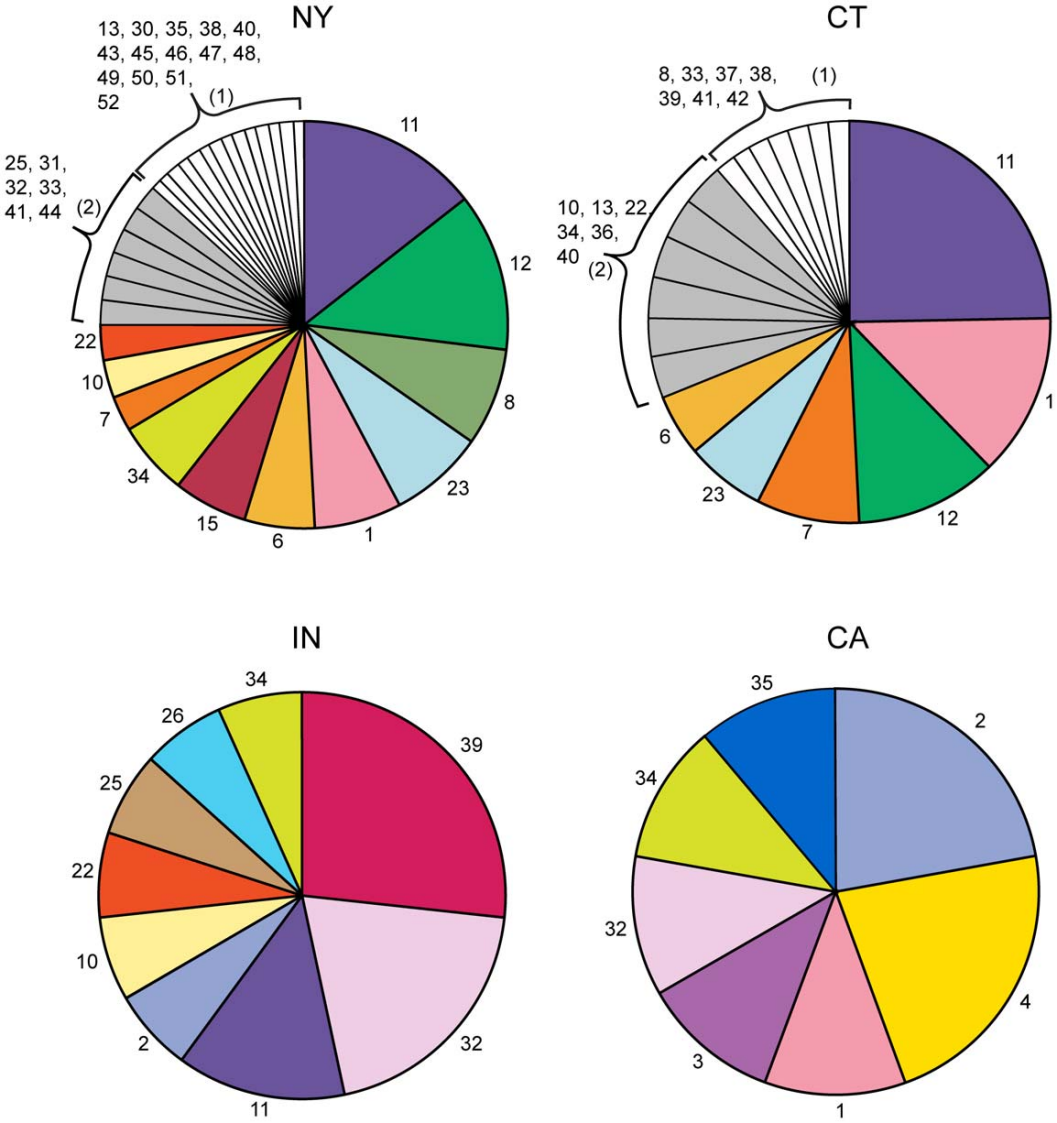
*B. burgdorferi* s.s. genotypes detected in NY, CT, IN, and CA. The number indicates the genotype and the pie size is relative to the proportion that the genotype was observed compared to all the genotypes in that region. The number of *B. burgdorferi* samples genotyped in each region was 104 for NY, 61 for CT, 14 for IN, and 9 for CA.

We were able to determine the genotypes of 139 of the 219 *B. burgdorferi* s.s. positive adult *I. scapularis* ticks. The remaining 80 samples contained either un-typable *B. burgdorferi* mixtures (n = 30), or there was insufficient DNA from the specimen to enable a full genotype analysis (n = 50). The predominant genotypes by region were: New York (8, 11, 12 and 23), Connecticut (11, 1, 12, and 7) and Indiana (39, 32 and 11) and represented 40%, 57% and 64% of the infected ticks, respectively (Figure 1). Nine of 15 *B. burgdorferi* s.s. positive adult *I. pacificus* ticks from Northern California were genotyped and found to predominately contain genotypes 2 and 4 (Figure 1).

### *B. burgdorferi* s.s. genotypic diversity by geographic region

**Figure 2:**
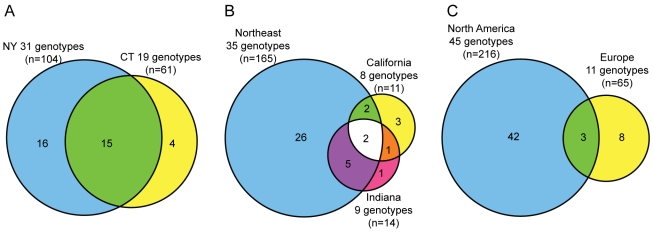
Venn diagrams displaying number of genotypes shared between (A) local, (B) regional, and (C) continental areas. The local and regional diagrams represent data from field-collected *Ixodes* ticks containing *B. burgdorferi* s.s. that were genotyped. The continental Venn diagram incorporates data from all samples (ticks and culture isolates) whose origin was known. The size of the circles is proportional to the number of genotypes in the region.

We compared the genetic diversity of *B. burgdorferi* in our field-collected specimens and culture isolates at the local level (New York vs. Connecticut), the regional level (Northeast vs. Indiana vs. California) and the continental level (North America vs. Europe), as shown in Figure 2A, B, and C respectively. Single specimens containing a resolvable mixture of genotypes were treated as separate counts for determining genotype frequency for the area.

In each region studied, a few genotypes were responsible for the majority of the *B. burgdorferi* infections (Figure 1). Proximal Connecticut and New York shared the highest percentage of common genotypes: 79% of CT genotypes were also observed in NY and 48% of NY genotypes were also observed in CT (Figure 2A). In examining North America, we grouped CT and NY (designated NE for Northeastern US) together due to their close proximity and the extent of shared genotypes. We found two genotypes (32 and 34) common to California, Indiana, and the Northeast (Figure 2B). Indiana shared 77% of its genotypes with the Northeast while 57% of California's genotypes were also found in the Northeast.

In the comparison of North American to European isolates (Figure 2C), only three genotypes were found on both continents. Of the *B. burgdorferi* s.s. genotypes observed in North America and Europe, only 6.6% of US genotypes (3 out of 45) were found in Europe and 27% of the European genotypes (3 of 11) were observed in the US.

### Characterization of genotypic *Borrelia* mixtures in *Ixodes* ticks

**Figure 3:**
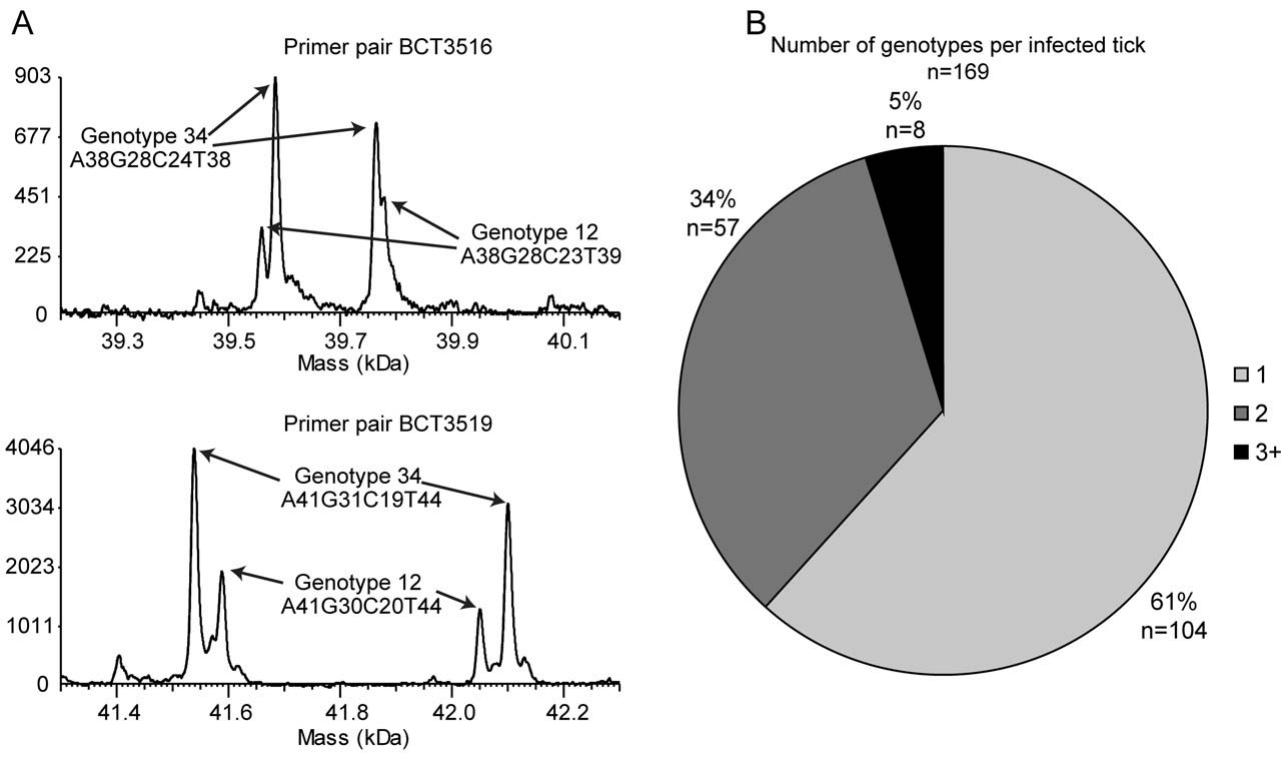
Detection of multiple *B. burgdorferi* genotypes in adult *I. scapularis* ticks. A) Example of assignment of alleles to a genotype in a mixture. Analysis of sample NY71 from a tick collected in Connetquot, Suffolk County, New York is shown. B) Proportions of the minimum number of *B. burgdorferi* s.s. genotypes per infected adult *Ixodes scapularis* tick from Indiana, Connecticut, and New York.

Of the 169 *B. burgdorferi*-positive adult *I. scapularis* ticks we characterized, 62% (n = 104) had a single genotype, 34% (n = 57) contained two genotypes and 5% (n = 8) had three or more genotypes (Figure 3). In those specimens with mixtures of more than one genotype, we were able to determine the individual *B. burgdorferi* genotypes in 52% of the specimens (34 of 65) based upon difference in the allele amplitude as described above in the methods section and shown in Figure 3A. Additionally, one of eleven *B. burgdorferi* infected nymphs from Suffolk County, NY was found to be infected with a mixture of two *B. burgdorferi* genotypes (genotypes 22 and 41). The proportion of *B. burgdorferi* s.s. genotypes per infected adult *Ixodes scapularis* ticks from Indiana, Connecticut, and New York is shown in Figure 3B.

### *Borrelia burgdorferi* s.l. genotyping of *I. ricinus* ticks from Southern Germany

178 *I. ricinus* ticks were tested for the presence of *Borrelia* on the genotyping assay and 36 were found to have *Borrelia* (20.2%). The majority of the positive samples were *B. afzelii* (n = 23) followed by *B. garinii* (n = 6) and *B. burgdorferi* s.s. (n = 3), *B. valaisiana* (n = 2), *B. lusitaniae* (n = 1), *B. spielmanii* (n = 1). In total 20 of the 36 samples positive for *Borrelia* were genotyped. The remaining samples contained un-genotypable mixtures or had limited levels of DNA.

Mixtures of *Borrelia* genotypes were also observed in the *I. ricnius* ticks with 8 (22.2%) of the 36 *Borrelia* positive specimens having two or more genotypes present. Five of the eight had a mixture of two genotypes while the remaining samples had 3 or more genotypes present. Three of the mixtures were resolved to their individual genotypes. Five of the mixtures were of *B. afzelii* genotypes, one a mixture of *B. garinii*, one was a mixture of *B. lusitaniae* genotypes, and one a mixture of *B. valaisiana*.

## Discussion

Lyme disease, caused by *B. burgdorferi*, has symptoms which are usually nonspecific, consisting of fever, fatigue, chills, headache, and muscle aches. In some cases, signs of the disease remain localized to an EM. In other cases there are signs of dissemination into systemic disease such as multiple erythema migrans lesions, central and peripheral neurologic abnormalities, cardiac impairment, and swelling of large joints. Different strains of *B. burgdorferi* have been associated with varying pathogenicities in the human host, with some strains more often observed in disseminated infections [10], [29]. Previous studies have shown that certain strains of *B. burgdorferi* are more likely to cause an erythema migrans and/or disseminate through the body[10], [30]. The ability to quickly identify the *Borrelia* species and associate particular genotypes with certain symptoms can have important patient-management implications. For example, the clinical observation of a single erythema migrans lesion where at least one of the recovered spirochetes is associated with an invasive phenotype might alert the treating physician to consider the use of an antibiotic, such as an intravenously administered one, that will reliably penetrate the central nervous system [31], [32] or, at the very least, the physician would monitor the patient for development of neurologic symptoms even with administration of oral antibiotics.

Previous studies have also shown culturing spirochetes from ticks or clinical specimens can alter the genotypes observed as compared to those obtained from direct PCR [33], [34]. Using our rapid multilocus PCR/ESI-MS genotyping assay, we have demonstrated that we can genotype *B. burgdorferi* directly from adult or nymphal ticks without the need for culture. Additionally, we have shown that our PCR/ESI-MS method can be used to detect and genotype *B. burgdorferi* directly from a clinical specimen. The PCR/ESI-MS genotyping assay has the added benefit of being able to identify the species and genotype a variety of *Borrelia* that can cause Lyme disease.

Studies have shown that the multilocus sequence typing (MLST) approach provides a finer separation of strains than does typing based on the *ospC* gene alone [15]. Using our multi-locus PCR/ESI-MS genotyping assay, several *ospC* groups were subdivided into multiple genotypes. In North America we observed 44 different genotypes of *B. burgdorferi*, 35 of which were found at sites within 100 miles of each other, which could indicate a much more diverse population of *B. burgdorferi* than previously described.

In this study, we found that *Borrelia* infection rates in adult *I. scapularis* ticks from the northeastern US were similar to those observed previously[4], [35], [36]; *B. burgdorferi* infection rates were relatively lower in Indiana and California. An earlier study utilizing an MLST approach reported that no genotypes were shared between the Northeastern United States and the Midwest (with Ohio being the dividing state) [37]. In contrast to this observation, we found two genotypes in all three regions of the United States. Although our sample sizes from the Midwest and California were lower, the presence of these genotypes in each region may indicate that there was a widespread expansion of a few clonal isolates across North America followed by subsequent local diversification.

Past studies employing either single-strand conformation polymorphism (SSCP) or reverse line blotting (RLB) have shown that ticks can be infected with more than one strain of *B. burgdorferi* [4], [38]. Due to the limits of DNA sequencing, MLST approaches cannot resolve mixtures without culture. Due to the capability of mass spectrometry to resolve mixtures, our PCR/ESI-MS genotyping assay could detect and frequently resolve specimens containing a mixture of more than one genotype. Of the 169 *B. burgdorferi* positive *I. scapularis* ticks characterized from the US, 39% contained two or more genotypes. Of the 36 *Borrelia* positive *I. ricinus* ticks from Europe, we found 22.2% containing more than one pathogenic species/member of *Borrelia burgdorferi* sensu lato complex. These findings indicate genotype mixtures in ticks are common in both Europe and North America. Our finding of a nymph (which has fed only once) with multiple genotypes and 5% of the adult ticks (which have fed twice), with three or more genotypes indicates that ticks can become infected with more than one genotype of *B. burgdorferi* from a single feeding. Further studies are needed to determine if certain combinations of genotypes are more likely to co-infect a tick or cause disease in humans.

In this study we developed a very high resolution *Borrelia* genoptying assay that can genotype directly from a variety of sample types and discriminate *Borrelia* mixtures. We found a diversity of genotypes across geographic regions. This capability to rapidly identify and genotype *Borrelia* may facilitate better management of patients with Lyme disease and provide a better understanding of the association of *Borrelia* genotypes and disease. Our findings also underscore the importance of collaborations between scientists with environmental and microbial ecology expertise [1], [12] along with those in health related research. While our findings, which were not designed to be comprehensive, provides the impetus and shows the feasibility to explore other areas where Lyme is being to reported such as the southern US and northern Mexico. Such studies can contribute to a broader epidemiologic assessment of the breadth of potential Lyme disease in North America. Future studies focusing on *Borrelia* genotypes in nymphs will further define their genotypic co-infection rates and determine if certain genotypes tend to preferentially co-infect. The findings here indicate that the diversity of genotypes in ticks known to cause Lyme disease is greater then previously described and that *Borrelia* may frequently exist as genotypic mixtures in the environment.

## Supporting Information

Table S1 Table showing the detected A, G, C, T nucleotides detected for the different genotypes.
(0.04 MB XLS)
